# Assessing and disclosing test results for ‘mild cognitive impairment’: the perspective of old age psychiatrists in Scotland

**DOI:** 10.1186/s12877-021-02693-x

**Published:** 2022-01-12

**Authors:** Stina Saunders, Craig W. Ritchie, Tom C. Russ, Graciela Muniz-Terrera, Richard Milne

**Affiliations:** 1 Centre for Clinical Brain Sciences, University of Edinburgh, Edinburgh, UK; 2 Brain Health Scotland, Glasgow, UK; 3 Alzheimer Scotland Dementia Research Centre, Edinburgh, UK; 4 Society and Ethics Research, Wellcome Connecting Science, Cambridge, UK; 5 Department of Public Health and Primary Care, University of Cambridge, Cambridge, UK

**Keywords:** Risk disclosure, Pre-dementia stages, Mild cognitive impairment (MCI)

## Abstract

**Background:**

Mild cognitive impairment (MCI) is a condition that exists between normal healthy ageing and dementia with an uncertain aetiology and prognosis. This uncertainty creates a complex dynamic between the clinicians’ conception of MCI, what is communicated to the individual about their condition, and how the individual responds to the information conveyed to them. The aim of this study was to explore clinicians’ views around the assessment and communication of MCI in memory clinics.

**Method:**

As part of a larger longitudinal study looking at patients’ adjustment to MCI disclosure, we interviewed Old Age Psychiatrists at the five participating sites across Scotland. The study obtained ethics approvals and the interviews (carried out between Nov 2020–Jan 2021) followed a semi-structured schedule focusing on [1] how likely clinicians are to use the term MCI with patients; [2] what tests clinicians rely on and how much utility they see in them; and [3] how clinicians communicate risk of progression to dementia. The interviews were voice recorded and were analysed using reflective thematic analysis.

**Results:**

Initial results show that most clinicians interviewed (Total *N* = 19) considered MCI to have significant limitations as a diagnostic term. Nevertheless, most clinicians reported using the term MCI (*n* = 15/19). Clinical history was commonly described as the primary aid in the diagnostic process and also to rule out functional impairment (which was sometimes corroborated by Occupational Therapy assessment). All clinicians reported using the Addenbrooke’s Cognitive Examination-III as a primary assessment tool. Neuroimaging was frequently found to have minimal usefulness due to the neuroradiological reports being non-specific.

**Conclusion:**

Our study revealed a mixture of approaches to assessing and disclosing test results for MCI. Some clinicians consider the condition as a separate entity among neurodegenerative disorders whereas others find the term unhelpful due to its uncertain prognosis. Clinicians report a lack of specific and sensitive assessment methods for identifying the aetiology of MCI in clinical practice. Our study demonstrates a broad range of views and therefore variability in MCI risk disclosure in memory assessment services which may impact the management of individuals with MCI.

**Supplementary Information:**

The online version contains supplementary material available at 10.1186/s12877-021-02693-x.

## Background

In cases of dementia due to neurodegenerative disease, the dementia syndrome is preceded by a preclinical phase marked by pathological changes with no outwardly symptoms and a prodromal phase characterised by relatively mild symptoms of cognitive decline in the absence of overt functional impact [1–5]. Over the years, there have been efforts to clarify what characterises these early states on the neurodegenerative disease spectrum and the associated risk of decline to dementia. A concept approximating that of mild cognitive impairment (MCI), benign senescent forgetfulness, was first introduced in the 1950s to describe individuals with specific cognitive characteristics living in a Canadian care home [6]. Since the late 1990s, MCI has been defined in formal diagnostic criteria and is now commonly referred to as MCI due to Alzheimer’s disease as per the NIA-AA guidelines [7, 8]. These guidelines describe three stages of disease pathology: preclinical, prodromal (MCI) and dementia stages, which together constitute the Alzheimer’s disease (AD) continuum. It can, however, be difficult for clinicians to establish where any individual sits on this disease continuum and their likely future trajectory, in part because individuals with substantial AD pathology may have no cognitive impairment and may never develop symptomatic dementia [9, 10]. Furthermore, quantifying AD pathology is challenging due to severely limited availability and routine testing for amyloid and tau biomarkers is in front line services.

The clinical presentation of MCI commonly includes an individual noticing changes in cognition, most frequently memory, without functional impairments which would impede daily living. The Winblad (2004) criteria for MCI involve impairment on cognitive tasks (self and/or informant reported); and/or evidence of decline over time on objective tasks; preserved basic activities of daily living (ADL) (minimal impairment on complex instrumental functions); and no dementia [11]. There has only been one disease-modifying therapy which has received FDA approval over the last two decades [12] and individuals with MCI are often a target population for AD clinical trials [13, 14] as this state of mild decline may offer a window for secondary prevention of dementia. This is reflected both in the UK-based Manchester consensus statement on MCI [15] as well as the American Academy of Neurology MCI practice guidelines [16]. However, pathological cognitive decline may be difficult to differentiate from normal age-related cognitive decline. For example, assessing an individual’s ability to carry out ADLs involves subjectivity due to cultural and societal norms. Moreover, recent evidence points to up to 30% of people attending a memory clinic with MCI may well have a functional cognitive disorder [17] often overlooked as a possible diagnosis [18].

Considering MCI as prodromal AD, the assessments are in line with clinical investigations for early AD, using neuropsychological tests and biomarker evidence to establish possible AD pathology. The hallmarks of AD pathology include amyloid-β plaques, neurofibrillary tangles in the brain, and neuroinflammation through glial activation [19]. Biomarker assessments may involve neuroimaging or obtaining fluids such as cerebrospinal fluid (CSF), blood or less so in clinical practice, saliva. MRI is used primarily to look for evidence of atrophy, particularly in the medial temporal lobe [20, 21]; emerging research has also identified changes in the hippocampal subfields as potential early MRI biomarkers [22, 23]. MRI can also be used to quantify white matter lesions [24, 25]. In contrast, PET scans can be used to detect areas in the brain with decreased neuronal activity as indexed by glucose metabolic reduction [26] or measure amyloid load [27, 28]. CSF may be obtained to assess levels of amyloid-β protein, tau and the ratio between these. Blood plasma and saliva are potentially less costly for detecting proteins associated with AD pathology but due to several limitations (such as lack of normative values or validity compared to more commonly used biomarkers), remain primarily research investigations [29–31].

Biomarker assessments for early AD pathology, however, have limited availability in UK memory clinics outside of research settings [15], and furthermore, have significant limitations due to their poor predictive value. Lifetime risks of dementia associated with elevated amyloid in adults aged 65 to 85 years have been calculated to be 13.8 to 29.3%, compared to lifetime risk estimates of 7.1 to 18.7% in similarly aged adults with non-elevated amyloid [32]. Moreover, there are no specific biomarkers for confirming the presence of MCI [29] as while abnormal results from assessments indicate higher risk of future dementia, they neither provide certainty of decline from MCI to dementia nor suggest when this may occur [33]. Delineating between normal ageing and different stages of decline is not categorical as evidence suggests cognitive decline may start 3–7 years prior to being identified as having MCI and 1–11 years prior to dementia [34]. Thus, while a review of 32 cohort studies reports conversion rates of MCI to dementia at 1 year follow-up between 10.2 to 33.6% (median: 19.0%) and at 2 years between 9.8 to 36.3% (median: 18.6%) [35], studies looking at longer term follo-ups concluded that most individuals with MCI had not converted to dementia at 4.5-year follow-up [36] or even at a 10-year follow-up [37, 38]. Other evidence suggests a 10–15% yearly conversion rate of clinic-based MCI patients [37].

There is thus considerable uncertainty over the prognostic value of MCI and unique challenges in identifying, managing and conveying to patients the prognosis of this condition. Though mild decline for some individuals may never progress further, for others MCI marks the first manifestation of AD. The potential for progression also has implications for clinicians working with the concept, and for patients and families. These include what it means for an individual to learn of having MCI and the risk of future deterioration in their brain health towards dementia, but also the nature of the clinical encounter in which this information is conveyed and discussed. While approaches to, and impact of, communicating a dementia diagnosis have been studied in detail [39–43], and there is growing evidence of how patients engage with the uncertainties associated with MCI [44, 45], the specificities of the clinical encounter and disclosure process for MCI have received less attention. Previous research focusing on communicating MCI has identified complexities associated with constructing the boundaries around MCI, AD and normal ageing in memory clinics from the clinicians perspective [46–48]; a wide variation in the clinical use of MCI from experts perspective [49] and limitations in the support services can offer to individuals without dementia [50].

In this paper, we report findings of qualitative research with senior Old Age Psychiatrists in Scotland conducted as part of an on-going longitudinal study assessing the impact of being disclosed of having MCI from the patient’s perspective. These interviews explored clinicians’ views on, and understandings of, the concept of MCI and their approaches to the identification, disclosure (of aetiology and prognosis) and management of MCI in a clinical setting and to communicating the risk of future dementia.

## Methods

We interviewed Old Age Psychiatrists at five participating sites in Scotland, UK. We used convenience sampling and recruitment was via email invitations among five National Health Service (NHS) memory assessment services and open to all Old Age Psychiatrists at the service. There are at least 18 memory assessment services in Scotland [51] and 64 practising Old Age Psychiatrists in Scotland (and 702 in the UK) [52].

Interviews were carried out over the telephone between November 2020 and January 2021. Due to COVID-19 [53] restrictions, the participants were consented remotely ahead of the interview, using the approved Informed Consent Form. Subsequently, participants sent the signed Informed Consent Form to take part in the study to SS who countersigned this written form and returned it to the participant. One clinician who was not an Old Age Psychiatrist was excluded from the analyses.

Interviews followed a semi-structured schedule (Table 1) focusing on the diagnostic process of MCI and how clinicians communicate the risk of future dementia. To protect the anonymity of the participants in a specialised field of medicine, we report only limited characteristics of the study sample. The study obtained ethics approvals from South East Scotland Research Ethics Committee 1 (reference number: 18/SS/0013) as well as local R&D approvals.

**Table 1:** Interview schedule for understanding clinicians’ views on MCI

**1.** Are you likely to use the term Mild Cognitive Impairment (MCI) with patients in your care? **2.** How do you describe the condition of MCI to your patients in terms of it being a diagnosis/label/description of symptoms etc.? **3.** When you inform a patient of their MCI status, what do you say to them? **4.** Do you refer to any evidence or conversion rates around the prognosis? **5.** Is that consistent in how you communicate about MCI with all your patients? If not, what influences your communication with different patients? **6.** What sort of responses do you commonly get from patients when you communicate about MCI? **7.** What investigations do you rely on when identifying MCI? **8.** How informative are current tests for MCI in your view?	

The interviews were audio-recorded and transcribed verbatim. First, we developed a coding framework by identifying all of the themes mentioned in categories related to [1] investigations carried out in suspected MCI; [2] what is being said to the patients when they are informed of MCI; and [3] the management plan for MCI. We analysed all the transcripts following a qualitative thematic approach [54], identifying themes in transcripts, and using these to revisit the entire dataset, establish whether and how the particular theme was mentioned or not mentioned as part of the clinician’s standard clinical practice, and finally refining and defining them (see Appendix 1 for detailed analysis framework). The initial identification of themes was conducted by SS, and a subset of interviews were reviewed and independently analysed by RM. These authors then met, reviewed and refined the analysis. Subsequently, to enable us to give a quantitative picture of clinical practice, we also converted some of the individual themes into categorical “Yes/No” variables (the “No” variable included responses where something was not part of standard practice but may be done if there was anything unusual about a particular patient). The descriptive statistics of the key findings are presented in frequencies.

## Results

A total of 19 Old Age Psychiatrists were interviewed (*n* = 13 male; *n* = 6 female). All participants were at a senior (consultant) staff grade working in standard UK NHS memory assessment services. This represents about 30% of the Scottish Old Age Psychiatry Consultants workforce (and 3% of the UK Old Age Psychiatry Consultants workforce). The interviews lasted between 12 and 45 min. These memory clinics receive the majority of referrals from General Practice of people with memory difficulties and/or suspected dementia.

### The entity of MCI

There were differences in whether participants use the term MCI at all with patients in their care and if they do, whether MCI could be considered a diagnosis or a description.

#### Diagnosis or a description

Most of the participants reported using the term *mild cognitive impairment* with patients (*n* = 16/19), with the ones not using the term considering it unhelpful due to a lack of clear prognosis. There was a clear division of views among those who use the term MCI about whether the category should primarily be considered a *diagnosis* (*n* = 8/16) or a *description* (*n* = 8/16). For example, one interviewee positioned MCI as a diagnosis that clearly exists in relation to a longer Alzheimer’s disease trajectory, and may in fact overlap with it:

*MCI is a diagnosis. I don’t want to distress people, MCI could be early Alzheimer’s as well, could be the beginning of Alzheimer’s, I don’t want to distress people, so I usually say, “At your age many people have MCI.”*

Participant 17

In this response, the identification of MCI as a diagnosis is closely tied to its ability to offer a prognosis, while at the same time being common to people of a similar age. In contrast, other interviewees rejected the label of a diagnosis in distancing their use of MCI from dementia:

*I don’t feel it’s a diagnosis, I feel it’s like a pre-dementia stage, patient has problems, they’re aware they’ve declined in a certain way, but the decline is not severe enough to give a diagnosis of dementia.*

Participant 19

*Their memory isn’t as good as you might expect, so there are some memory deficits there that I see but you know I don’t think it’s that awful that I would be diagnosing them with a dementia.*

Participant 9

*I don’t think MCI is really a thing. I wouldn’t call it a diagnosis as I’d say there’s a certain criteria that needs to be met for a diagnosis, namely there’s a predictive value needed. But as I say, MCI has poor predictive value so no, I wouldn’t use this term.*

Participant 1

Another participant emphasised that a descriptive quality was intrinsic to the MCI concept, with implications for how patients were able to use the term.

*It’s just putting a technical term on a complaint that they [patients] present with. It doesn’t actually add anything to their knowledge about the condition or how to manage it.*

Participant 2

However, the distinction presented in these extracts was not necessarily firm or consistent even for a specific participant – for example, the extract below shows how, for some, the use of one or the other of ‘diagnosis’ or ‘description’ depends on the specific patient:

*I tend to describe it as a description of their difficulties, sometimes I may say diagnosis, I would say it’s sitting between normal ageing and dementia, using diagnostic label is driven by patient preferences – some patients want a label, what is the diagnostic name for this condition, some people are not really interested in the label and just want a description.*

Participant 20

For others, the concept of MCI either had limited value or had an inherent “wooliness” that necessitated a caveat that MCI is not a “proper” diagnosis. Instead, it was again defined by the absence of the alternative diagnosis, dementia.

*Majority of the time I use the term MCI if they don’t fill the criteria for dementia but have some symptoms. I mean, it’s not, it’s a bit woolly, the concept is not well defined.*

Participant 7

*I find it slightly meaningless in a clinical practice perspective, I think that patients would find it even more pointless. It’s just putting a technical term on a complaint that they present with. It doesn’t actually add anything to their knowledge about the condition or how to manage it.*

Participant 3

#### Confidence in conveying MCI

The diversity in views on MCI’s status as a diagnostic label are reflected in a broad range of views on how confident participants felt in the diagnostic process. The majority of the participants (*n* = 13/19) said that, when considering MCI, they are either not confident in ruling out early dementia, or question the value of the MCI label for the patient when it has inherent uncertainties. However, six out of the 19 participants said they felt confident it was the correct diagnosis at the time. Over half of the participants (*n* = 11/19) were not sure what would help diagnostic certainty, whereas *n* = 8/19 participants said their confidence would increase with access to tests with better specificity to tell apart MCI and dementia.

*If I’m not confident I might say to the patient that it’s quite possible that it’s already dementia but I’m just not sure. There are times I may say, let’s just call it MCI for now.*

Participant 14

*Case by case basis, sometimes you call it that but suspect it may be dementia but too early.*

Participant 15

*MCI would be the least confident of all the different diagnostic categories I deal with. Diagnostically it’s often seen as a temporary bay, my confidence increases, when I’ve seen someone with MCI a year later and a year later.*

Participant 20

### Assessing MCI

**Fig. 1:**
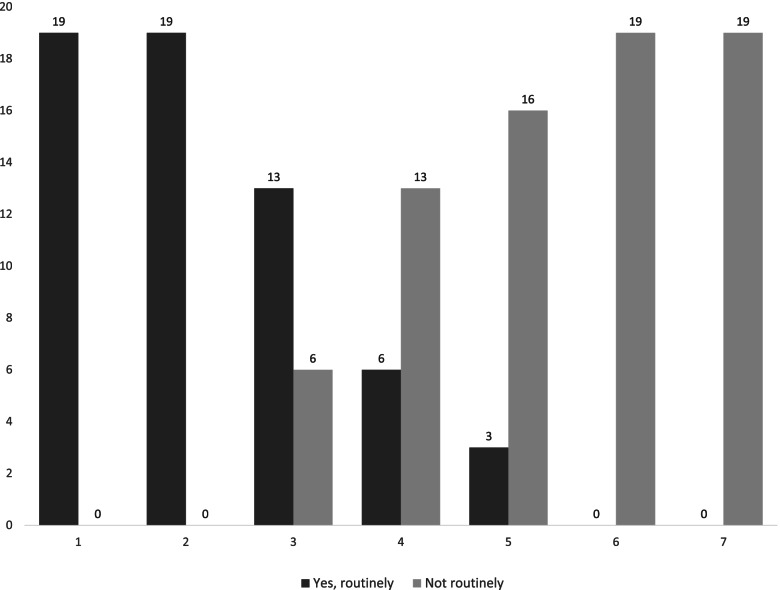
Routine investigations carried out in MCI: [1] Clinical history including functional assessment, [2] Global cognitive assessment (ACE III), [3] Neuroimaging, [4] Referral to neuropsychological assessment [5] Occupational Therapy assessment, [6] C SF testing, [7] PET imaging for any ligand (FDG/Amyloid/DAT)

Assessing MCI was considered a clinical judgement and the majority of the participants (*n* = 13/19) considered a thorough clinical history (including functional assessment) the most important component in the diagnostic process. There was variety in how many additional investigations the participants mentioned carrying out as standard practice when suspecting MCI (Fig. 1): the largest number of respondents (*n* = 9/19) carry out three investigations; *n* = 5/19 participants carry out two investigations; *n* = 3/19 carry out five investigations; *n* = 2/19 carry out four investigations.

One significant critique of MCI was the diagnostic tools available are not sensitive or specific enough for AD or other neurodegenerative pathology. More specifically, participants felt some – such as those assessing functioning – were too subjective. Additionally, participants critiqued MCI for having poor predictive value (and therefore getting patients worried), and being poorly defined.

*Without a brain to look at under a microscope, you know how are we really making these decisions, it’s about being aware that we can make our best assumption and we can take good history and we can do good examinations, but how good are they? I think a lot of old age psychiatrists feel, it’s not very… where is the evidence base for it, the hard and fast [evidence]?*

Participant 5

Participants’ thinking specifically focussed on the value of neuroradiological assessments. Most of the participants (*n* = 16/19) considered neuroradiology (primarily CT scans) to have limited clinical utility due to the reports being too non-specific. There was acknowledgement that while such scans may be reassuring for patients, for clinicians they can be less reassuring as the results are difficult to tell apart from normal ageing:

*The scan reports are not helpful as they are not specific enough, nearly all of them say vascular changes.*

Participant 1

*Majority of CT brain scans come back either fairly normal or no specific changes.*

Participant 16

As such, their role in the diagnostic process was necessarily secondary to clinical judgement.

*Scans … they support the diagnosis, they don’t make the diagnosis. When they support your diagnosis you’re pleased, when they don’t you think, oh well it’s not terribly relevant.*

Participant 14

However, as one participant elaborated, the lack of specificity of neuroimaging was not necessarily a quality of the scans themselves, but also of the multidisciplinary clinical relationships related to requesting and interpreting scans:

*The quality of reports is variable, they often say no tumours, major strokes etc as opposed to a very refined neuroradiologist who might have an interest in dementia might give a better report of the same image. I don’t think we have good quality service in terms of imaging. It boils down to the interest of the reporting neuroradiologist.*

Participant 20

### Communicating about MCI

**Fig. 2:**
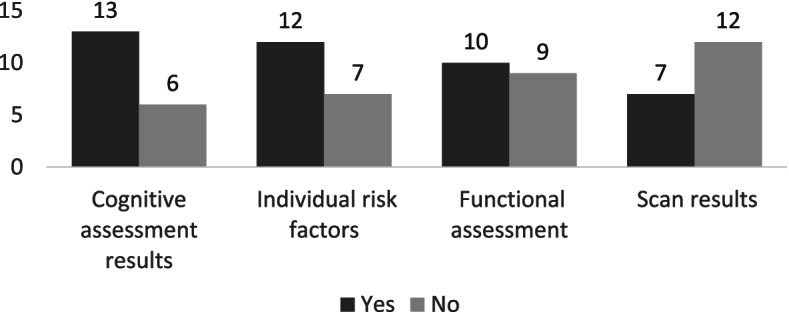
Specific investigations referred to when disclosing MCI as standard practice

There was a range in how many investigations the participants referred to as standard practice when discussing MCI with patients (Fig. 2): the largest number of respondents (*n* = 6/19) refer to three investigation in their explanations; *n* = 5/19 participants refer to two investigations; *n* = 3/19 refer to no investigations; *n* = 2/19 refer to one investigation; *n* = 2/19 refer to four investigations and finally one participant refers to five investigations (the fifth one being blood test results performed by the General Practitioner). When describing MCI to patients, the majority of the participants (*n* = 13/19) reported referring to memory. Fewer participants (*n* = 5/19) reported referring to the brain. Just over half of the participants (*n* = 10/19) mentioned referring to a disease continuum with normal ageing on the one side and the dementia syndrome on the other (with one participant using visual aids to demonstrate the continuum); and nearly half of the participants (*n* = 9/19) refer to what would be the expected norm for the patient’s age. Similarly, nearly half of the participants (*n* = 9/19) discuss specific areas of concerns the patient has; and under half (*n* = 8/19) explicitly mentioned discussing medications the patient is taking.

Participants described the importance of acknowledging people’s concerns about their cognitive complaints, and the value of seeking clinical advice:

*[I say] “I’m glad that you came to discuss this, because what you’ve told me we were also able to measure in the assessment. It’s difficult to know what’s going to happen with these problems.” I would acknowledge their cognitive impairment and the severity.*

Participant 3

#### The absence of dementia

In terms of how participants approached this conversation with patients, participants described highlighting that MCI is *not* a diagnosis of dementia:

*I flag up that we need to monitor this over time, but let’s be positive about the fact that this does not fall into the dementia category, but we need to keep an eye on this.*

Participant 4

*All I’m saying when I say you have MCI is that you don’t have dementia. Yes, there is a possibility this might progress or not, we would be more than happy to review things if things declined.*

Participant 12

*I would say it’s not dementia, it’s a condition called MCI*

Participant 20

#### The possibility of future dementia

This negative definition of MCI as *not* dementia at the moment, however, also involves acknowledging uncertainty about whether it may become dementia in the future. As one participant put it:

*“I always think of MCI as a threat of dementia hanging over you. Almost like a breast lump which you worry about, wonder if the next breast lump is going to develop into carcinoma.”*

Participant 13

All participants reported conveying uncertainty around what to expect in the future. Nearly half (*n* = 10/19) of the participants discuss prognosis and nearly half (*n* = 9/19) refer to any evidence regarding the stability or conversion to dementia in MCI. The conversion rates participants referred to varied between 5 and 50%. Under half of the participants (*n* = 8/19) said they assess the expectations of how much the patient would like to know, although no specific measure for assessing patient’s expectations was mentioned. Nearly half (*n* = 9/19) said their communication may vary between different patients. Aside from patients’ expectations this was primarily influenced by the patient’s perceived anxiety, education levels and the presence and contribution of a family member.

Discussing their approach to this conversation, participant 20, elaborating on the comment above, described how they would state that:

*There is a clear deficit in memory problems … in a significant proportion it remains stable, in some people it will decline, we don’t know who is going to stay stable and who is going to decline, and we will check this in a year’s time, how things are progressing.*

Participant 20

For the participant below, this was easily summarised as:

*I tend to say there are three groups of people: [those] who improve, stay the same or decline.*

Participant 2

Another, however, delivered a similar message in a way that emphasised that these three categories are themselves stages on a continuum between ‘floating’ and ‘sinking’:

*As you get older, the memory gets worse, for some people they may struggle a little bit more, but they are floating just underneath the water but not at the bottom of the lake where you find people with dementia who really struggle with their daily activities, engaging with their commitments. I’d say you’re just below the waterline, you might get used to struggle like this or you might get better or that you might develop dementia in the next few months, at the moment we don’t know.*

Participant 7

**Fig. 3:**
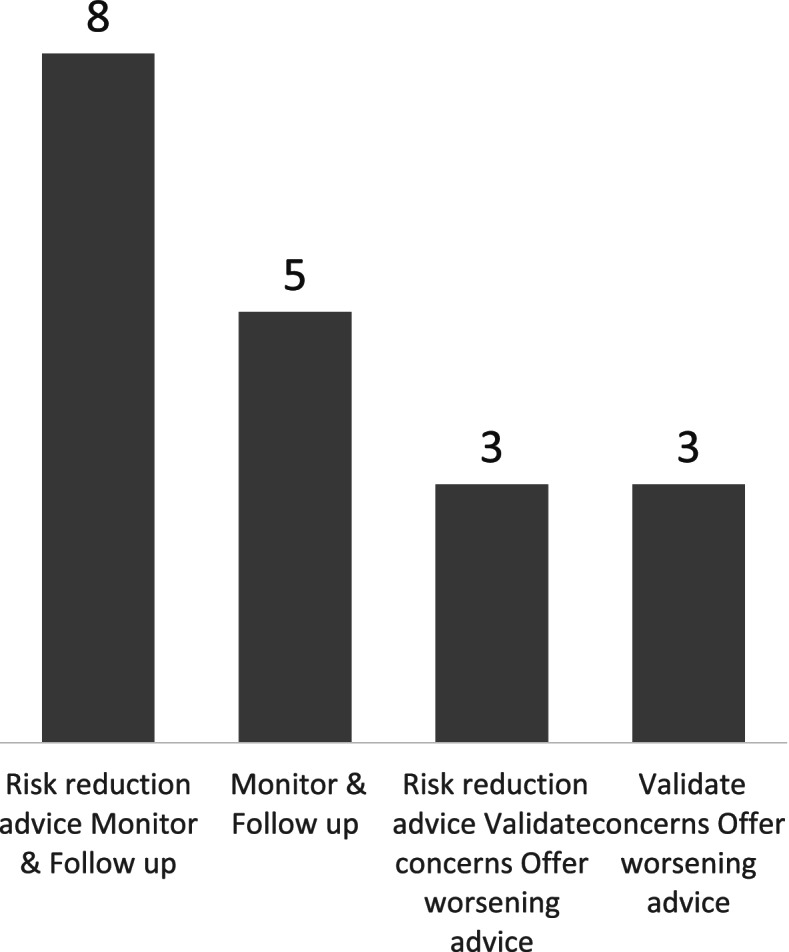
Standard management plans for MCI: [1] Risk reduction advice, Monitor & Follow up, [2] Monitor & Follow up, [3] Risk reduction advice, Validate concerns, Offer worsening advice, [4] Validate concerns, Offer worsening advice

As the quotes above introduce, the uncertainty about future progression introduces consideration of what future care looks like. Four categories for management of MCI among the study participants could be identified (Fig. 3). In terms of discharge from the service, over half of the participants (*n* = 13/19) keep the patients in the memory clinic system and offer a routine follow-up appointment either 12 months later (*n* = 7/19) or between 6 and 12 months later (*n* = 6/19). Under one third of the participants (*n* = 6/19) discharge patients with MCI back to the GP with no routine follow-up appointment by the service. Regardless of whether follow-up appointments are offered as a standard, all participants report giving patients advice to return if or when there are further concerns. More than half of the participants (*n* = 11/19) reported offering advice for risk reduction (Fig. 3). Under half of the participants (*n* = 8/19) said written information is either handed to the patients or displayed in the waiting rooms.

### Patient responses to being informed of having MCI

Finally, participants were asked to consider how patients responded to being informed that they had MCI. Over half of the participants (*n* = 11/19) reported a positive *response* from patients, with fewer (*n* = 6/19) saying that they detected anxiety in some patients about being at risk of developing dementia.

Among positive responses participants reported relief associated with the absence of a dementia diagnosis:

*I get from people a sense of relief, “so you’re not diagnosing me with dementia and that’s a positive.”*

Participant 4

*Patients are different, some take it very easy and are delighted, they are not too bothered, other people might be a little worried. I don’t see people getting very worried that they’re getting dementia, majority are actually relieved that they have MCI rather than dementia.*

Participant 7

However, this sense of relief related to the responses above – here the idea of “having MCI” as a diagnosis. Similarly, another participant described how:

*It is certainly my impression that it’s validating them that there is a condition that we are calling MCI.*

Participant 10

Other participants also emphasised the importance of validation and, further, the actions associated with this, particularly a management plan.

*I would say for the vast majority it was reassuring that there wasn’t enough to diagnose dementia and … more importantly that we were going to keep an eye on things.*

Participant 5

*I think they’re probably quite happy and relieved I’m not diagnosing them with dementia. And I think they’re usually quite relieved that I say I will follow them up within a year.*

Participant 9

## Discussion

The findings from our study illustrate the heterogenous nature of the condition of MCI as it is used in routine clinical practice. Even among a specialised field of senior clinicians, we found remarkable differences in attitudes and approaches to MCI. We found diversity in perspectives on how MCI is understood as a diagnostic category and variability in the identification, disclosure and management of MCI in a clinical setting. This resulted in differences in communication about MCI, with variation in how prognosis and risk were discussed with patients as standard clinical practice. In the UK, dementia services are run under a predominantly psychiatric model of care with a phenomenological and symptomatic focus [55]. This was also reflected in the results of our study with many participants describing the core of their job in diagnosing dementia – and the concomitant engagement with MCI as ‘not dementia’.

In the USA, MCI practice guidelines advise on the assessment and management of MCI [16]. However, in the UK there are no specific clinical guidelines for managing MCI, although the ‘Manchester Consensus’ proposal [15] makes a number of recommendations including equitable access to clinical and biomarker assessments. In our work, we identified a combination of investigations carried out as standard clinical practice, with all participants doing a clinical interview (including a functional assessment) and a global cognitive assessment but a varying number of participants also relying on a detailed neuropsychological assessment, neuroimaging or an OT assessment. More than half of the participants tended to refer patients to neuroimaging and even fewer tended to refer patients to neuropsychological testing as standard practice. Study participants were, however, sceptical about the clinical utility of neuroimaging as well as to a lesser extent, neuropsychological testing.

In the UK there is extremely limited access to biomarker assessments in standard clinical practice [14]. Although the latest revision to NICE clinical guidelines introduced the possibility of CSF sampling as an option if AD is suspected [56], no participants in our study mentioned this as part of their standard clinical practice. While such testing may also contribute to identifying patients, who may be eligible candidates for clinical trials targeting biomarker changes associated with early Alzheimer’s disease, offering research opportunities was not explicitly mentioned by participants in our study results. This finding is consistent with other research looking at clinicians’ communication about MCI, for example a study in Dutch memory clinics where access to research was also not mentioned as part of the disclosure process [48].

More than half of the study participants offer individualised risk reduction advice as part of standard practice, which is in line with the Manchester consensus’ recommendations. This follows evidence that approximately 40% of dementia cases could be prevented by targeting 12 modifiable risk factors [2]. The Manchester consensus also recommends annual follow up of individuals identified as having MCI either by primary care or secondary care services which was in line with the results of this study where more than half of the participants offered follow up as standard practice though all participants reported giving worsening advice.

Diversity of approach was also evident in the range of ways participants discussed prognosis. Half of the participants discussed possible decline rates to dementia and the other half of the participants preferred not to introduce any figures for probability of decline (the latter including participants who reported not using the term MCI at all). Comparatively, this is less than what was reported by clinicians in a US study looking into MCI disclosure where over half of the clinicians mentioned discussing dementia risk when disclosing MCI [57], but more than Dutch clinicians in the aforementioned study by Visser and colleagues (2020) who rarely mentioned risk of dementia [48]. Regardless of the differing approaches to disclosure of MCI, the majority of the participants in our study reported observing positive responses from patients who had been informed they have MCI. Although several participants acknowledged that in some cases, patients may have anxiety around the prognosis of MCI and fear of decline to dementia. This finding is consistent with a study which concluded that European clinicians were less likely than American clinicians to consider MCI disclosure as unnecessary worry to patients and family [58]. In neurological disorders, some suggest that not diagnosing is preferable to falsely labelling individuals with obscure or non-existent diseases [59]. Although MCI may develop into dementia, there is considerable uncertainty about whether dementia will manifest and thus if the “holding” label of MCI is either appropriate or indeed, serves a useful purpose.

The results of our study illustrated participants’ reasoning of whether the condition of MCI constitutes a diagnosis, resulting in a near even split between those who consider it a diagnosis and those who do not. However, the majority of the participants in our study reported using the term MCI, in line with previous surveys of UK Old Age Psychiatrists and suggesting that MCI is widely used as either description or diagnosis in UK memory clinics [60]. The use of a diagnostic label may be manifold and nuanced. By definition, a medical diagnosis aims to improve clarity and communication, provide a focus for treatment, inform prognosis, and in some cases, may be useful for preventative treatments [61]. For patients, a diagnosis can enable the social incorporation of the person who has an illness, and allows for an explanation of differences [62]. The results of our study though, highlight a contradiction whereby the majority of participants reported that the term MCI provides neither clarity around communication nor necessarily informs prognosis. Looking forward, it may be the case that MCI will become less relevant as a clinical category as *maintenance of brain health* through risk reduction across the life course and along a continuum of brain ageing becomes more widely adopted.

One of the limitations of this qualitative study looking into clinicians’ views on MCI is relying on self-reported practices rather than observing the disclosure process in situ. We have therefore framed the results of the study around practices mentioned by the participants as standard practice rather than what was evidenced by observation. Another limitation is a small sample size though this is in keeping with other qualitative work around clinicians and experts’ views on the condition of MCI. Our results represent better the Scottish Old Age Psychiatry Consultants workforce but are limited in scope in terms of the UK. A small sample size enabled the use of semi-structured interviews which allowed for an open discussion and invited the participants to offer insights which are most important for them. The homogenous sample of this study may be limiting in offering a narrow perspective through the lens of Old Age Psychiatrists, but it may also be considered a strength of the study because the results may reflect more closely wider approaches to MCI within this medical speciality in the UK. We consider the systematic approach to the data collection and analysis a strength of this study.

Finally, this paper concentrates solely on the views and experiences of clinicians. However, the Manchester consensus [15] recommends that research should also examine the psychosocial impact of being diagnosed with MCI on patients and carers. This is the focus of the wider work of which the present study is a part.

## Conclusions

MCI is a state that is open to diverse clinical interpretation, leading to variations in diagnostic testing, clinical management, and communication to patients even within a small geographical area and single health system. There is variation in the communication of test results, prognostic certainty, and risk of further decline. Accordingly, the results of our study examining the views of Old Age Psychiatrists showed a range of opinions around the clinical usefulness of the term MCI.

Our results provide further evidence that the condition of MCI is complicated due to the limited translation into practice of the knowledge we are gathering of early AD pathology. This is because the availability and knowledge around biomarker test accuracy in the prodromal AD stage is still limited. This is compounded clinically as there is no guidance in the pre-dementia stages regarding symptoms and functions against normative population data on ageing.

In the future, bringing the evidence presented in the current study together with detailed research on patients’ perspectives and experiences of the MCI diagnostic process would provide valuable input into recommendations for MCI disclosure.

## Supplementary Information

**Additional file 1.**

## Data Availability

The datasets generated and analysed during the current study are not publicly available due to the interviews being drawn from a relatively small pool of expert interviewees and as such may be difficult to entirely de-identify for public sharing but are available from the corresponding author on reasonable request.

## Abbreviations

AD: Alzheimer’s Disease
ADL: Activities of Daily Living
CSF: Cerebrospinal fluid
CT: Computer tomography
COVID-19: Coronavirus disease (COVID-19)
GP: General practitioner (family doctor)
MCI: Mild cognitive impairment
MRI: Magnetic resonance imaging
NHS: National Health Service (UK)
NIA-AA: National Institute on Ageing and Alzheimer’s Association
NICE: National Institute for Health and Care Excellence (UK)
OT: Occupational therapy
PET: Positron emission tomography
R&D: Research & Development
