# Correction to: Assessing and disclosing test results for ‘mild cognitive impairment’: the perspective of old age psychiatrists in Scotland

**DOI:** 10.1186/s12877-022-02770-9

**Published:** 2022-02-28

**Authors:** Stina Saunders, Craig W. Ritchie, Tom C. Russ, Graciela Muniz-Terrera, Richard Milne

**Affiliations:** 1 Centre for Clinical Brain Sciences, University of Edinburgh, Edinburgh, UK; 2 Brain Health Scotland, Glasgow, UK; 3 Alzheimer Scotland Dementia Research Centre, Edinburgh, UK; 4 Society and Ethics Research, Wellcome Connecting Science, Cambridge, UK; 5 Department of Public Health and Primary Care, University of Cambridge, Cambridge, UK

**Correction to: BMC Geriatr 22, 50 (2022)**

**https://doi.org/10.1186/s12877-021-02693-x**

After publication of this article [1], the authors reported that in the section “Background”, third paragraph, the first sentence was incorrectly given as “Considering MCI as prodromal MCI, the assessments are in line with clinical investigations for early Alzheimer’s disease dementia,…” and should have read “Considering MCI as prodromal AD, the assessments are in line with clinical investigations for early AD,…”. Moreover, Fig. 1 did not match with the figure legend and axis description. The figure should have appeared as shown below.

**Fig. 1:**
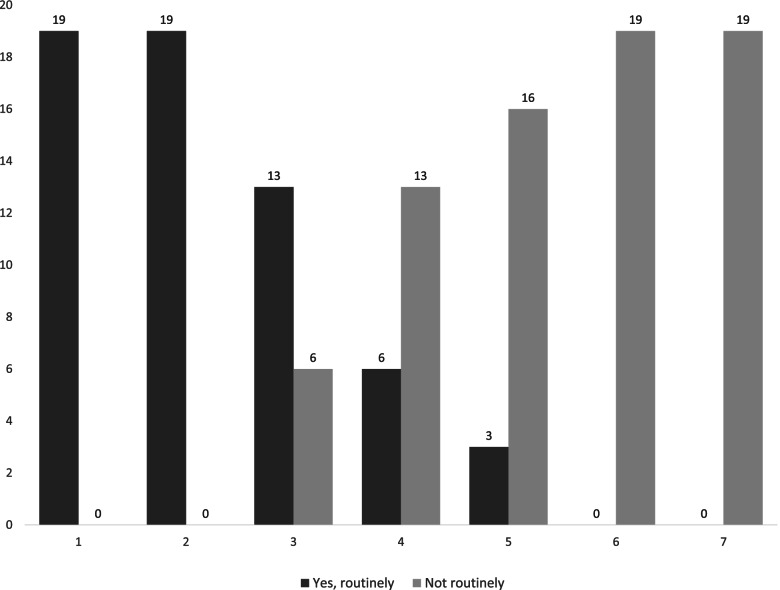
Routine investigations carried out in MCI: [1] Clinical history including functional assessment, [2] Global cognitive assessment (ACE III), [3] Neuroimaging, [4] Referral to neuropsychological assessment [5] Occupational Therapy assessment, [6] C SF testing, [7] PET imaging for any ligand (FDG/Amyloid/DAT)

The original article [1] has been updated.

## References

[1] Saunders S, Ritchie CW, Russ TC (2022). Assessing and disclosing test results for ‘mild cognitive impairment’: the perspective of old age psychiatrists in Scotland. BMC Geriatr.

