# Supplementary material for: Assessing and disclosing test results for ‘mild cognitive impairment’: the perspective of old age psychiatrists in Scotland
Source: BMC Geriatr. 2022 Jan 12;22:50. doi: 10.1186/s12877-021-02693-x (PMC8754072; doi:10.1186/s12877-021-02693-x)
Supplement: Supplementary file 1 — Additional file 1. [file 12877_2021_2693_MOESM1_ESM.docx]

**Appendix 1**

**Analysis coding framework**

Top level analysis groups form the basis for reporting, with quantification based on the number of responses to the individual lower-level items.

- Diagnostic status of MCI
  - Diagnosis
    - Prognostic
    - Shared
  - Description
    - Limited value
  - Patient-centered variation in terminology
- Clinical assessment
  - Clinical judgement
    - Primacy of clinical judgement vs assessments
    - Clinical relationships
  - Investigations mentioned
    - Clinical history
    - Global cognitive
    - Neuroradiology
      - Unclear value
      - Patient vs clinician value
    - Neuropsychology referral
    - OT assessment
    - CSF
    - PET imaging
- Clinical communication
  - Investigations discussed with patients
  - Framing of MCI
    - Defining the term
      - Memory
      - Brain
      - Continuum
      - Age-related norms
    - Distinguishing from dementia
      - Reassurance
      - Review
        - Plans for discharge or follow-up?
    - Prognosis and future progression
      - Uncertainty
      - Discussion of prognosis?
      - Discussion of risk reduction?
      - Variation by patient expectations?
  - Patient-specific concerns
    - Acknowledging importance of seeking clinical advice
  - Medication
- Reported patient perspectives
  - Reported emotional response
    - Positive
      - Relief
      - Validating concerns
      - Follow-up
    - Negative
      - Anxiety
